# Health state dependent multiphoton induced autofluorescence in human 3D *in vitro* lung cancer model

**DOI:** 10.1038/s41598-017-16628-3

**Published:** 2017-11-24

**Authors:** Vasyl Kilin, Christophe Mas, Samuel Constant, Jean-Pierre Wolf, Luigi Bonacina

**Affiliations:** 1 GAP, University of Geneva, 22 chemin de Pinchat, CH-1211 Geneva 4, Switzerland; 2 OncoTheis Sàrl, 18 chemin des aulx, CH-1228 Plan-les-Ouates, Geneva Switzerland; 3 Epithelix SAS, 219 Rue Laszlo Biro, 74160 Archamps, France

## Abstract

Lung diseases pose the highest risk of death and lung cancer is a top killer among cancers with a mortality rate up to 70% within 1 year after diagnosis. Such a fast escalation of this cancer development makes early diagnosis and treatment a highly challenging task, and currently there are no effective tools to diagnose the disease at an early stage. The ability to discriminate between healthy and tumorous tissue has made autofluorescence bronchoscopy a promising tool for detection of lung cancer; however, specificity of this method remains insufficiently low. Here, we perform autofluorescence imaging of human lung cancer invading a human functional airway using an *in vitro* model of Non Small Cell Lung Cancer which combines a reconstituted human airway epithelium, human lung fibroblasts and lung adenocarcinoma cell lines, OncoCilAir™. By using two-photon laser induced autofluorescence microscopy combined with spectrally resolved imaging, we found that OncoCilAir™ provides tissue’s health dependent autofluorescence similar as observed in lung tissue in patients. Moreover, we found spectral and intensity heterogeneity of autofluorescence at the edges of tumors. This metabolic related heterogeneity demonstrates ability of tumor to influence its microenvironment. Together, our result shows that OncoCilAir™ is a promising model for lung cancer research.

## Introduction

Lung cancer is the most common cancer-related cause of death worldwide^1–3^. Detection of lung cancer in its early stages significantly increases the chance of survival, but it remains a challenge at present^1,4–6^. One of the most promising techniques is autofluorescence bronchoscopy. Most tissues fluoresce under excitation of a suitable wavelength, and this fluorescence is frequently used as a readout to study various biological processes by monitoring fluorescence from naturally occurring fluorophores such as collagen, elastin, melanin, keratin, porphyrins, NAD(P)H and flavins^7^. NAD(P)H and flavins are the most contributing to autofluorescence. These molecules are involved in multiple metabolic reactions, which are expected to differ among cancer and healthy tissue^8^. It has been reported that in many cancers, tissue exhibits a weaker autofluorescence and red shifted spectrum^9–12^.

In lung cancer dysplasia, carcinoma *in situ* and microinvasive carcinoma tissues exhibit much weaker green fluorescence and slightly weaker red fluorescence than normal tissues when excited at 380 to 440 nm^12,13^. Also, clinical studies of the healthy bronchial wall, and metaplastic and dysplastic bronchial lesions showed a significant decrease in the autofluorescence intensity and red shifted autofluorescence from the lesions compared to healthy bronchial tissues^14–16^. These differences in fluorescence of healthy and cancerous tissue provides an attractive contrast for the detection of tumors and has triggered the development of autofluorescence bronchoscopy^4,17–19^. Several endoscopic autofluorescence systems have been developed such as SAFE (by Pentax, Tokyo, Japan), D-Light (by Karl Storz GmbH, Tuttlingen, Germany), DAFE (by Richard Wolf GmbH, Knittlingen, Germany) and LIFE (by Xillix Technologies, Vancouver, BC, Canada). All are commercially available and widely used in clinical studies for the detection and localization of abnormal tissue by autofluorescence^20,21^.

Autofluorescence methods allows identification of pre-invasive lesions and has potential for postoperative surveillance^22^. Treatment of such lesions is expected to significantly increase efficiency in the curing and diagnosis of lung cancer^23,24^. However, currently the specificity of this method is at best moderate, ranging from 50%^25,26^ to 63%^27^ and many ongoing clinical trials are aimed at justifying this technique for the detection of lung cancer. One of the possible direction to improve autofluorescence bronchoscopy is using multiphoton excitation, because of its advantages such as deeper penetration depth, reduced photodamage, and lack of out-of-focus bleaching^28–30^. This has fostered the development of multiphoton endoscopes with great potential in clinical research for tissue imaging^31–35^. However, the development of endoscopy techniques is slowed down by the fact that it is almost impossible to perform an accurate autofluorescence study of human tissue within the lungs due to the laborious procedure involved. While in most cases tissue health state influence autofluorescence the exact difference of spectral parameters might vary significantly. This variation might be partly explained by heterogeneity of NADH and flavins autofluorescence caused by altered cellular metabolism in different patients as well as response to treatment, therefore one needs to establish a large database to determine the long-term outcome of patients undergoing autofluorescence bronchoscopy^20,36^.

Currently, autofluorescence bronchoscopy is used in combination with conventional white light reflection bronchoscopy^26^ and afterwards, biopsies are taken from areas considered abnormal for further examination. Both, morphological and spectral anomalies are used to identify the tumors. While studies performed on biopsies are most relevant to humans, they are limited by the lifetime span of the sample ~5 days. Moreover, the diagnostic selectivity of bronchoscopy strongly depends on lesion size and for lesions <3 cm the selectivity varies in range 14–50% and could be more than 50% for lesions > 3 cm implying that lung cancer tends to be detected at an advanced stage^37–40^. Also, because of these reasons, until now, there has been no accepted explanation of the differences in fluorescence for normal and cancerous tissue in the lungs^4,41,42^.

To address these issues and open questions, multiple lung cancer modelling systems are used. The most common system is 2D plated isolated cells, such as non-small cell lung cancer (NSCLC), which accounts for approximately 85 percent of all lung cancers^43,44^. 2D systems allow for the study of molecular processes and drug assessment, but they are inadequate for the development of effective therapies for lung cancer. More sophisticated models also have been proposed, such as NSCLC based tumor spheroid^45–47^, microfluidic chip-based 3D co-cultures^48–50^, *ex vivo* 3D lung cancer models based on a decellularized matrix^51,52^, patient derived xenograft models^53^, and precision-cut lung tumor biopsies^54,55^. The fundamental limitation in these models is their inability to mimic the tumor’s microenvironment, which has significant consequences on growth features and responses to therapies^45,56^. This is a result of loss of tumors’ ability to construct the surrounding tissue to promote their own growth and progress, and to change both molecular and mechanical signals coming from the adjacent healthy environment. Similarly, fundamental differences in human and animal models limit their application as preclinical cancer models, which compromises the design of efficient cancer therapies^2,57–59^. To minimize such differences, new tools to manipulate the genome in widely used mouse model are in development^60^. Therefore, in such models, autofluorescence will be strongly dependent on origin of used components and will not fully represent autofluorescence from human lungs tissue. In addition, ability to reproduce cellular microenvironment is of significant importance, because it will modulate cells metabolism^61–63^. Thus, model capable to fulfill these requirements is essential for accurate autofluorescence study of lungs tissue.

**Figure 1:**
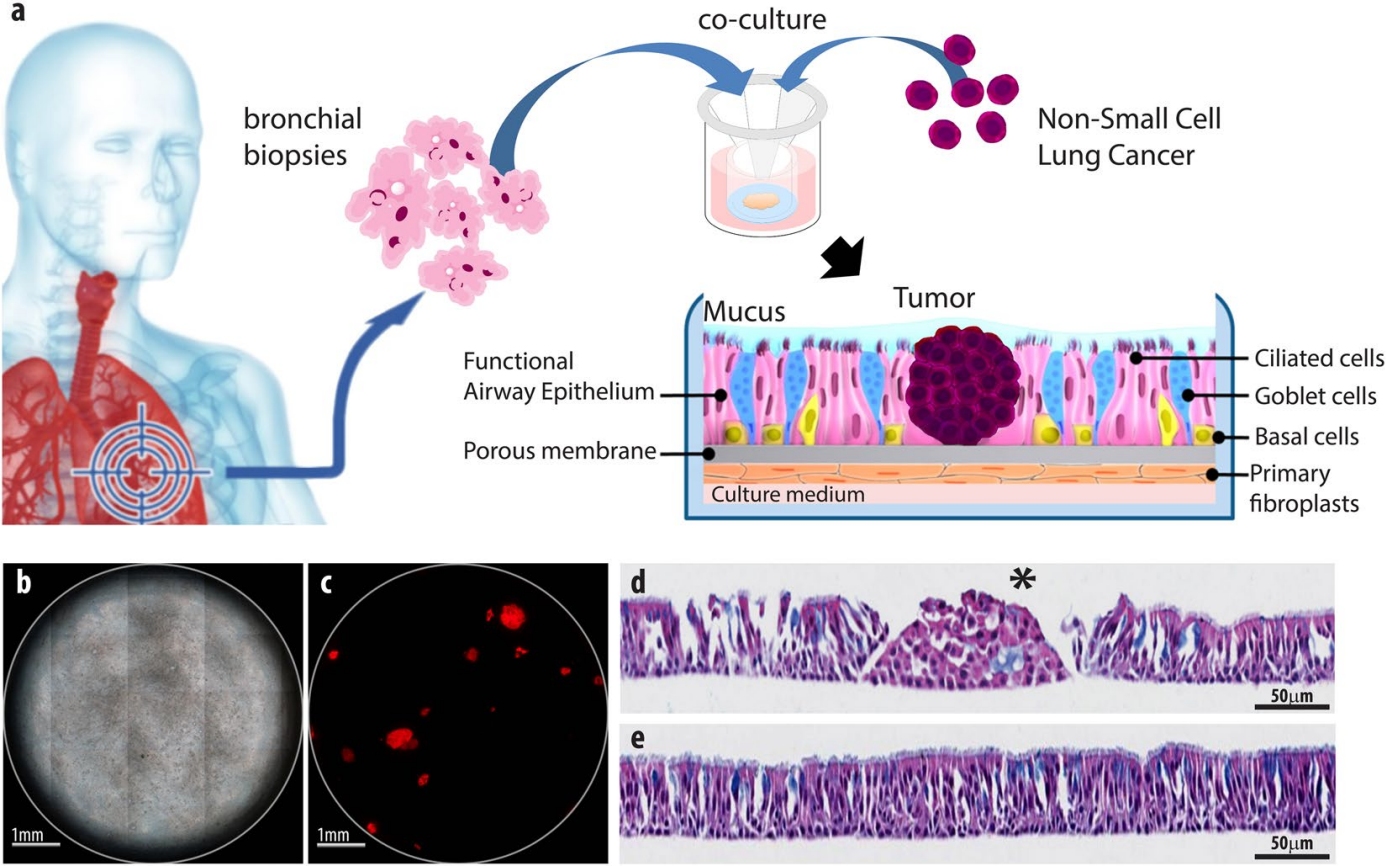
The OncoCilAir™ model. (**a**) Schematic representation of lung cancer invading a human functional airway modelled by OncoCilAir™ tissue cultured in Transwell® inserts. (**b**) Phase contrast and (**c**) fluorescence images showing a human airway epithelium with EGFR tumor nodules (mRFP labeled) reconstituted at the air–liquid interface in a Transwell® insert. Hematoxilin eosin histological staining shows the proper differentiation of the healthy region of the airway (**e**) with a pseudostratified columnar epithelium containing ciliated cells, goblet cells and basal cells closed to the microporous membrane and cluster of non-polarized tumor cells (d, star) invading the epithelium.

In this work, we performed autofluorescence imaging of lung tumor using our recently developed model which reproduces a tumor invading human lung tissue - OncoCilAir™ ^2,56^. This cellular model is based on the co-culture in Transwell® insert at the air-liquid-interface of bronchial cells and lung fibroblasts reconstituted from primary human airway epithelial cells and NSCLC cell lines. After inducing appropriate differentiation, the system closely reproduces lung cancer nodules invading a human functional airway epithelium (Fig. 1). This functional respiratory epithelium is comprised of ciliated cells with active cilia, goblet cells secreting mucus, basal cells, fibroblasts and tumor nodules. The OncoCilAir™ model was already demonstrated to closely mimic biologically relevant processes *in vivo* and it has been used successfully as a predictive tool for anticancer drug evaluation^2,56^.

Such combination of human cells and suitable bioscaffolds results in a long lifetime (up to 4 months) model accessible for accurate correlation of autofluorescence with health state of the tissue at the earliest stages of cancer. For this purpose, we performed two-photon laser-induced autofluorescence microscopy of lung cancer tissue modelled by OncoCilAir™. Using two-photon excitation and spectrally resolved imaging, we first demonstrated that autofluorescence observed from this model possesses the same spectral characteristics as previously reported in autofluorescence bronchoscopy. This autofluorescence is strongly tissue health dependent exhibiting twice as stronger intensity in healthy tissue compared to cancerous tissue. From known structure of OncoCilAir™ and spectrally resolved microscopy, it follows that this intensity contrast could be adequately explained by differences in amounts of endogenous fluorophores in tumors. In addition, we found that autofluorescence intrinsic of healthy tissue is observed at the edges of tumor body and it decays towards its center over 150 μm. In the same direction the autofluorescence intrinsic of cancerous tissue increases. Such a modulation of autofluorescence inside the tumor indicates that OncoCilAir™ models tumor ability to influence its surrounding healthy tissue. Understanding such phenomena is essential for development of new treatments which will prevent spreading of cancer in healthy tissue. Combined, our findings show that the OncoCilAir™ model mimics the autofluorescence response from the human tissue of healthy and cancerous lungs, opening up new possibilities for accurate studies of lung cancer development with a great promise for autofluorescence bronchoscopy improvements.

## Material and Methods

### Human lung cancer 3D cultures

OncoCilAir™ is a complex cellular model based on the co-culture of three different human components: bronchial cells, lung fibroblasts, and NSCLC cell lines. The resulting tissue consists of a functional respiratory epithelium which comprises developing tumor nodules. Lung fibroblasts were derived from small bronchi explants cultured in DMEM supplemented with 10% fetal calf serum, seeded onto inverted 33 mm^2^ Transwell® inserts (Ref Costar #3470, Cambridge, MA) and allowed to attach for 3 h at 37 °C. The Transwell® inserts were then turned back and transferred to 24-well plates. The respiratory epithelium was reconstituted from primary human airway epithelial cells (hAECs) isolated from patient bronchial tissue samples by enzymatic digestion and grown in an airway culture medium (EP04MM from Epithelix, Switzerland). Briefly, 2.5 × 10^5^ hAECs were plated on top of the porous membrane undercoated with fibroblasts. NSCLC cell lines, such as HCC827, were incorporated in each insert of the 24-well plates at different cell concentrations (0.05–1 million cells per well) to obtain the optimal seeding density required for the formation of tumors nodules. For tissue with tumors expressing mRFP, NSCLC cells were tagged with a lentivirus expressing the red fluorescent protein (mRFP). Two days after seeding, hAECs were switched to air–liquid interface (ALI) for at least 25 days to obtain differentiation into a ciliated pseudostratified airway epithelium. Airway cells were obtained from patients undergoing surgical lobectomy. All human biopsies were obtained according to the local ethical committee requirements (Commission cantonale d’éthique de la recherche scientifique de Genève [CCER]). Experimental protocols have been approved by CCER. Experimental procedures were explained to patients, and all of them signed an informed consent.

### Histology

Tissues cultures, 30 days *in vitro*, were rinsed in PBS and fixed by immersion in 4% formaldehyde for 30 min. Fixed tissues were embedded into paraffin, sectioned at 5 µm and processed for staining with Hematoxilin Eosin.

### Adenocarcinoma cell line 2D cultures

HCC827 cells, carrying an EGFR tyrosine kinase domain deletion, were grown at a density of 3 × 10^4^ cells/cm^2^ in RPMI-1640 culture media (R0883, SIGMA) supplemented with 10% fetal calf serum.

### Two photon laser induced autofluorescence microscopy

Autofluorescence imaging was performed on a Nikon multiphoton inverted microscope (A1R-MP) coupled with a Mai-Tai tunable Ti:sapphire oscillator from Spectra-Physics (100 fs, 80 MHz, 700–1000 nm). A Plan APO 20 × WI N.A. 0.75 objective was used to focus the excitation 720 nm and to epi-collect the autofluorescence signal. The collected signal was processed by a Nikon A1 descanned spectrometer. The signal was collected and directed through an optical fiber to the spectral detector, where it was diffracted by a grating and projected on a 32-PMT array. The working range in detection was 400 nm to 650 nm and the resolution was set to 10 nm for a total of 25 independent detection channels.

To obtain 3D hyperspectral images, we performed autofluorescence imaging with sequential refocusing to acquire focal stack using 720 nm for excitation. Hyperspectral images were obtained by acquiring 25 images of the sample’s spectrally resolved signal at the same time. This allows to get a spectrum (of 25 points) for each pixel of the images.

Spectral unmixing was performed considering measured emission signal in pixels as a sum of autofluorescence spectra of healthy and cancerous tissue, with additional contribution from mRFP protein and from porous nitrocellulose membrane. We identified 4 individual spectral components: autofluorescence from healthy tissue and autofluorescence from unlabeled HCC827 cells plated in 2D, mRFP fluorescence from mRFP tagged HCC827 cells plated in 2D and autofluorescence from polyethylene terephthalate (PET) membrane. Tissue and PET membrane autofluorescence was recorded using 720 nm excitation. mRFP fluorescence was acquired using 1000 nm excitation in order to obtain autofluorescence-free mRFP spectrum. The spectral unmixing procedure provides the weights of each spectral component. These weights are then used for construction of spectrally unmixed 3D images.

Acquisition, rendering and spectral unmixing were performed using NIS-Elements software.

## Results and Discussion

### Human lung cancer modeled by OncoCilAir™

To build fully functional human lung airway tissue, respiratory epithelium was reconstituted from primary human airway epithelial cells (hAECs) isolated from patient bronchial biopsies by enzymatic digestion. Next, hAECs cells were plated on top of porous membrane undercoated with fibroblasts. Finally, NSCLC cells were incorporated with a subsequent switching of hAECs cells to an air-liquid interface, thereby inducing differentiation into a ciliated pseudostratified airway epithelium. After full differentiation, OncoCilAir™ possesses several important features. First, all its components are of human origin, and the model mimics human tumor-stroma interactions to assess therapies targeting host-tumor interactions. Second, the flexibility of the system allows choosing cell lines mutated in different oncogenes (EGFR, KRAS) for building its tumor component, thus simulating patient stratification^2^. Third, it includes both cancerous and healthy tissues, introducing the possibility of experimenting simultaneously drug efficacy and local toxicity within a single culture. Fourth, it can mimic the tumor’s microenvironment specific to one patient, a primary requirement for personalized medicine. Fifth, it reproduces the essential protective features of the lungs, such as mucus and cilia beating allowing mucociliary transport. The possibility of accessing these protection mechanisms in a conventional way has enormous potential for the development of inhalation based nanomedicine delivery^64–66^. Finally, its long lifespan (up to 4 months) permits the study of tumor development and evaluating chronic treatment^2^. All together, these properties are critical for autofluorescence studies as they participate to define the tissue autofluorescence response.

### Autofluorescence and mRFP fluorescence from NSCLC cells plated in 2D cultures

**Figure 2:**
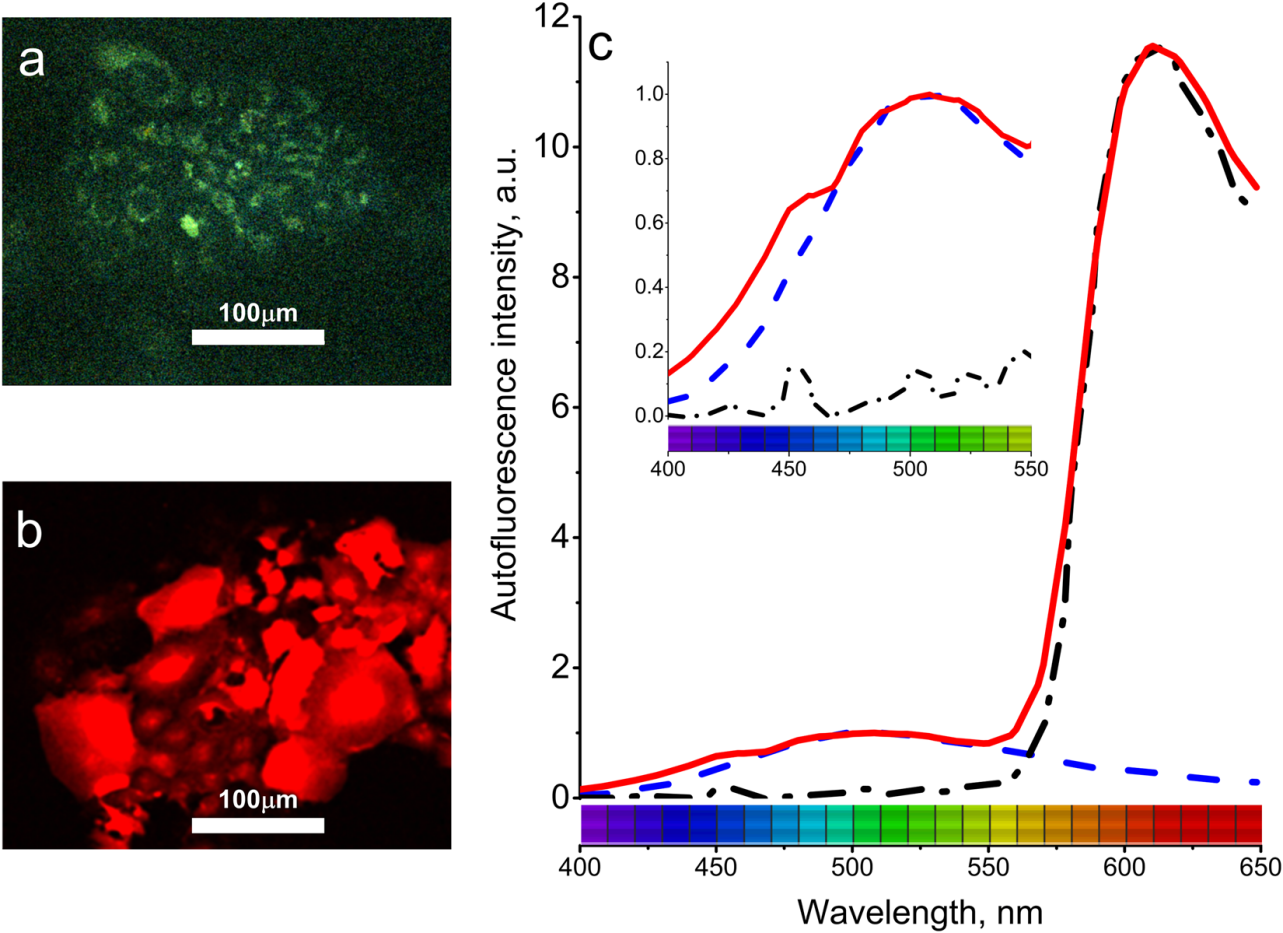
(**a**) HCC827 cells plated in 2D and imaged by autofluorescence induced at 720 nm. (**b**) HCC827 expressing mRFP imaged using 720 nm excitation. (**c**) Autofluorescence spectra of cells without mRFP – blue dashed line; simultaneous recording of autofluorescence and mRFP fluorescence using 720 nm excitation – red solid line. mRFP fluorescence excited at 1000 nm – black dash dot line. Inset: close-up of the autofluorescence spectra in 400–550 nm region.

In order to localize tumors in tissue and to study them via autofluorescence microscopy, we tagged NSCLC cells with a lentivirus expressing red fluorescent protein (mRFP). This protein could be excited by two-photon absorption from IR laser of wavelength 720 nm or 1000 nm and emits at 610 nm^67^. The 720 nm excitation wavelength allows to excite endogenous fluorophores which has absorption at 360 nm through two photon absorption and allows simultaneous acquisition of tissue autofluorescence and mRFP spectra. To establish signal crossover in detection channels that occurred because of spectral overlap between fluorophores, we performed spectral imaging of NSCLC cells plated in 2D not expressing (Fig. 2a) and expressing (Fig. 2b) mRFP. The intrinsic autofluorescence spectra of cancer cells was observed with the strongest intensity in the range of 490–510 nm.

By 1000 nm excitation, we determined the autofluorescence-free spectrum of mRFP-tagged NSCLC cells and then applied 720 nm excitation to induce autofluorescence and mRFP fluorescence simultaneously (Fig. 2c). By comparing the acquired spectra, we found that the autofluorescence and mRFP fluorescence spectra had minimal overlap and was adequate for simultaneous identification of tumors using RFP signals along with autofluorescence study of tissue.

### Autofluorescence microscopy of human lung cancer modeled by OncoCilAir™

We next performed autofluorescence hyperspectral imaging with sequential refocusing to acquire a focal stack of 2D spectral images and then assembled them into 3D images. The healthy tissue was observed to be 50 µm thick and autofluorescence from layers composed from cilia, goblet and basal cells were comparable with autofluorescence from primary fibroblasts.

**Figure 3:**
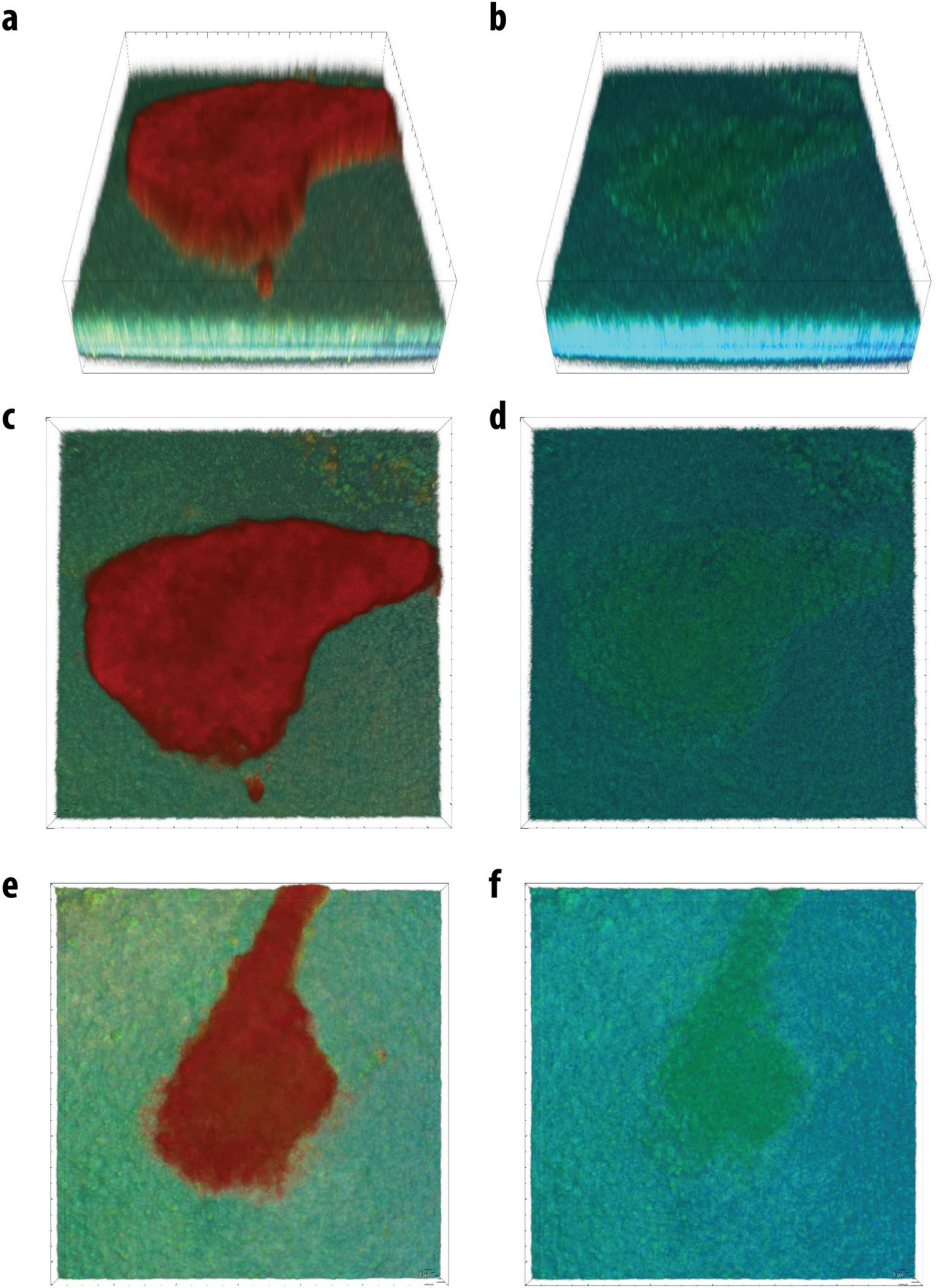
Typical hyperspectral images of tumors expressing mRFP (red) in healthy lung tissue (green-yellow) modeled by OncoCilAir™ from the same patient. (**a**,**c**,**e**) Tumors in shown using full spectral response 400–650 nm. (**b**,**d**,**f**) Tumors shown using spectral response limited to 400–550 nm. Images were obtained with 720 nm excitation. The box size is 1270 × 1270 × 180 $\mu m$. (**a**,**c**,**b** and **d**) The same tumor shown from a different point of view.

**Figure 4:**
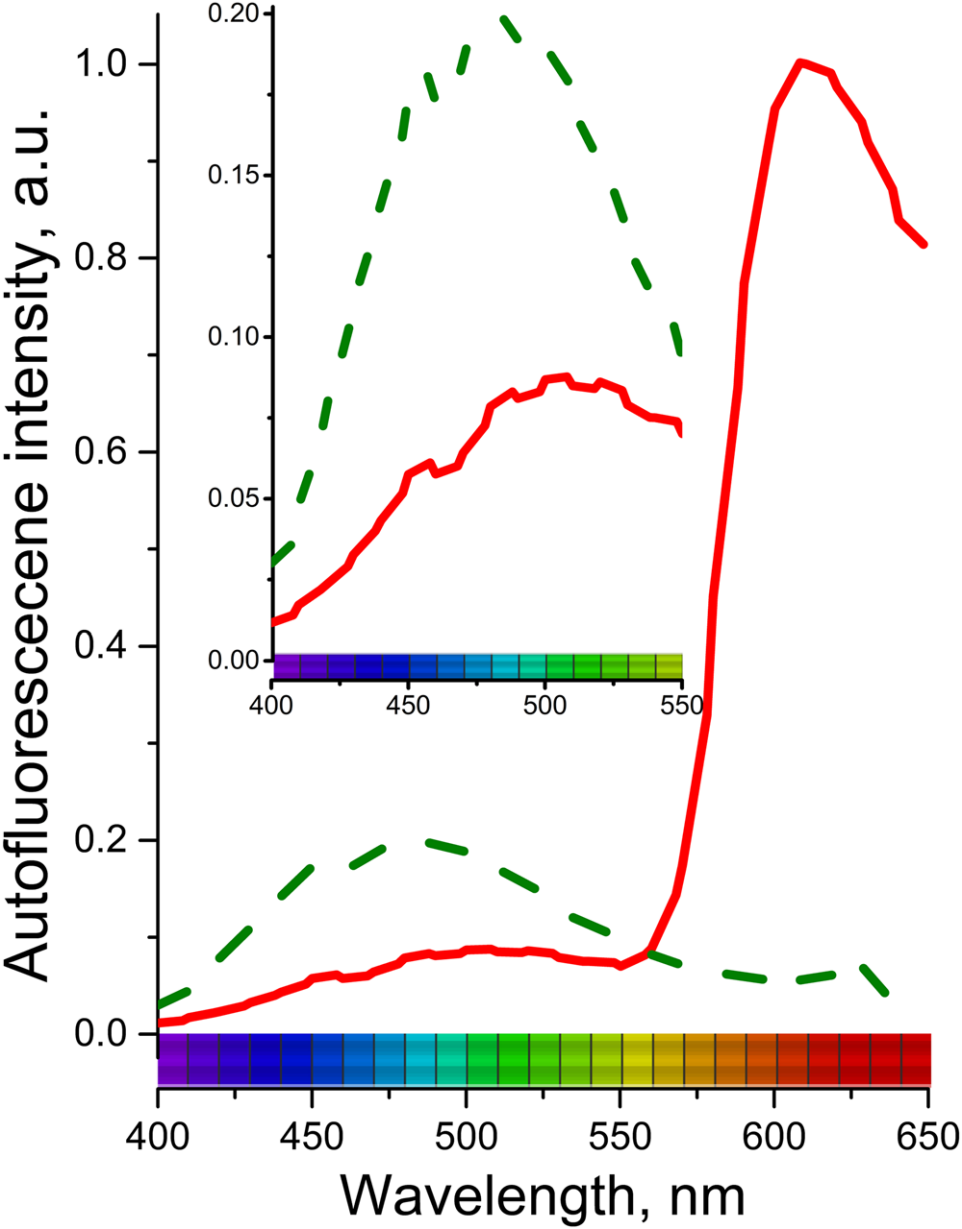
Autofluorescence spectra from human lung tissue modeled by OncoCilAir™ induced at 720 nm; healthy tissue – green dash- line; cancerous tissue – red solid line; Inset: close-up of autofluorescence in 400–550 nm region.

Tumors were typically observed as nodules with characteristic sizes ranging from 100 µm to 1 mm growing in healthy tissue (Fig. 3). Under 720 nm wavelength excitation, we observed that healthy tissues exhibited autofluorescence that was typical for NADH-rich cells with a maximum in a region 470–490 nm (Fig. 4). On the contrary, tumors were characterized by much weaker autofluorescence (Figs 3 and 4). The intrinsic autofluorescence from tumor tissue was seen to differ in both spectral shape and intensity of the fluorescence emission - maximum intensity was in the 500–510 nm region and was observed up to two times weaker compared to healthy tissue (Fig. 4). These results are in agreement with clinical *in vivo* studies, which showed that bronchial lesions exhibit a significant decrease in autofluorescence intensity and spectral red shift from the lesions compared to healthy bronchial tissues^13–16^ and with autofluorescence emission from fluids of patients with and without lung cancer^68^. This indicates the ability of the OncoCilAir™ model to mimic both autofluorescence of healthy and cancerous lung tissues.

Although health state-dependent fluorescence differences were thought to be a result of increased epithelial thickness, increased blood flow and/or a reduced concentration of fluorophores in abnormal tissue, there is currently no consensus in the literature^41,42^. From our results, which were obtained on tissue where no epithelial thickness changes or blood flow modulations were observed, one can conclude that intensity and spectral contrast in human airway epithelium might be partly ascribed to a decreased concentration of endogenous fluorophores or their quenching.

Taking into account recent *in vivo* fluorescence lifetime studies that have demonstrated no significant lifetime difference in autofluorescence from healthy and cancerous human lungs tissue^69^ (previous work reported 9% lifetime contrast^70^), we would rather exclude quenching. Therefore, health-dependent autofluorescence in human airway epithelium might be sufficiently explained by metabolic-related variations in endogenous fluorophores. The absence in OncoCilAir™ of epithelial thickness alternation and increased blood flow enable to perform an accurate study of metabolic related spectrum differences of cancer and healthy tissue.

### Heterogeneity of autofluorescence inside tumors

**Figure 5:**
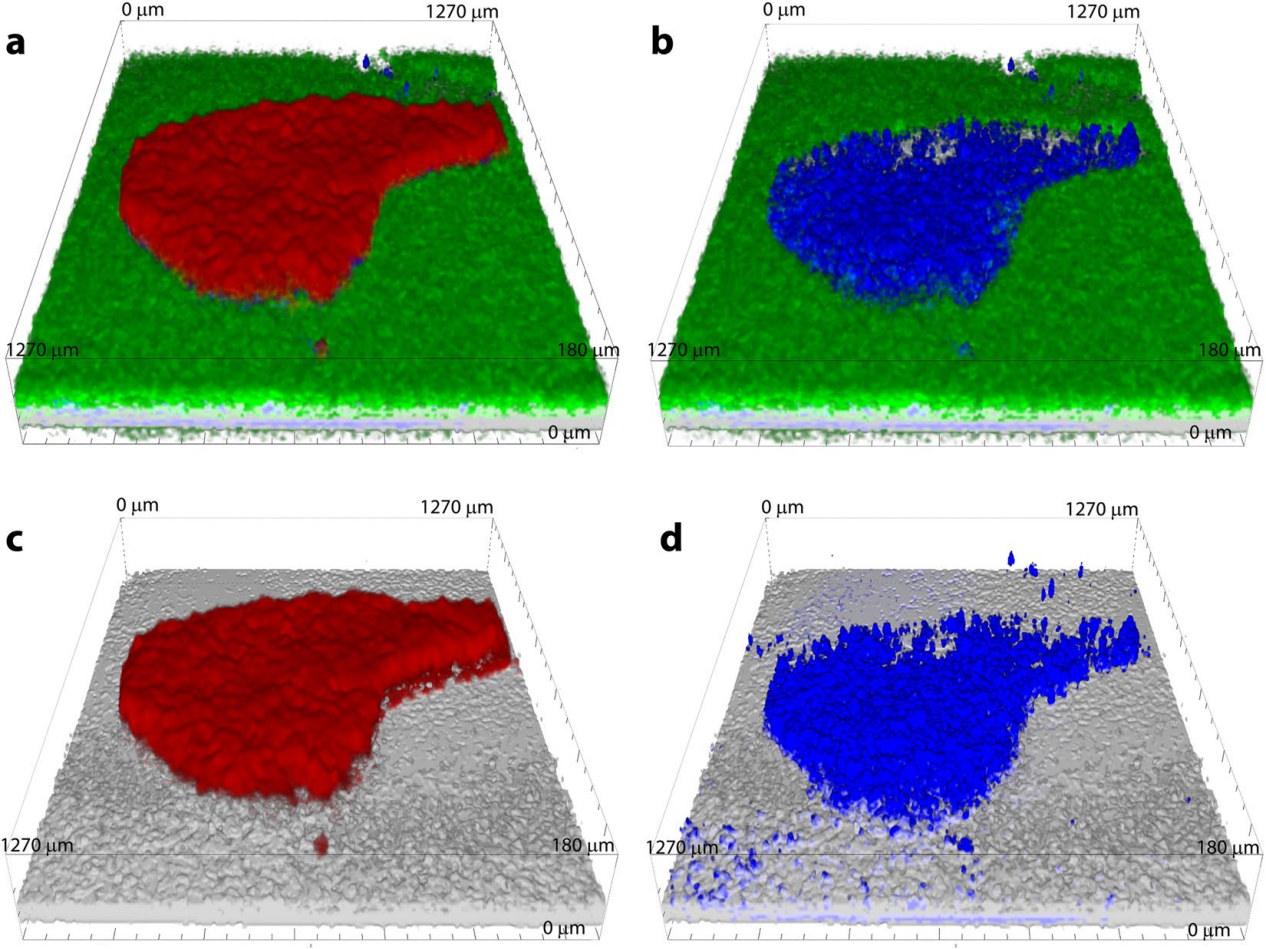
Spectrally unmixed images of tumors expressing mRFP modeled by OncoCilAir™. (**a**) Superposition of all unmixed components; (**b**) healthy tissue component (green) and tumor (blue) identified by autofluorescence; (**c**) tumor localized by mRFP fluorescence; (**d**) tumor localized by autofluorescence. Membrane shown in grey.

To investigate the morphological properties of autofluorescence in tumors and to quantify intensity differences in autofluorescence between healthy and cancerous tissues, we performed spectral unmixing. Spectral unmixing was performed considering measured emission signal in pixels as a sum of autofluorescence spectra of healthy and cancerous tissue, with additional contribution from mRFP protein and from porous PET membrane. As result of spectral unmixing, we obtained the weights of each individual spectral component. Next, we used these weights for construction of intensity-based images (Fig. 5). In such representation weights indicate localization of endogenous fluorophores specific for healthy tissue and cancerous tissue (green and blue respectively Fig. 5b and d). And similarly, localization of mRFP protein and PET membrane in the tissue (red and gray respectively Fig. 5a and c).

**Figure 6:**
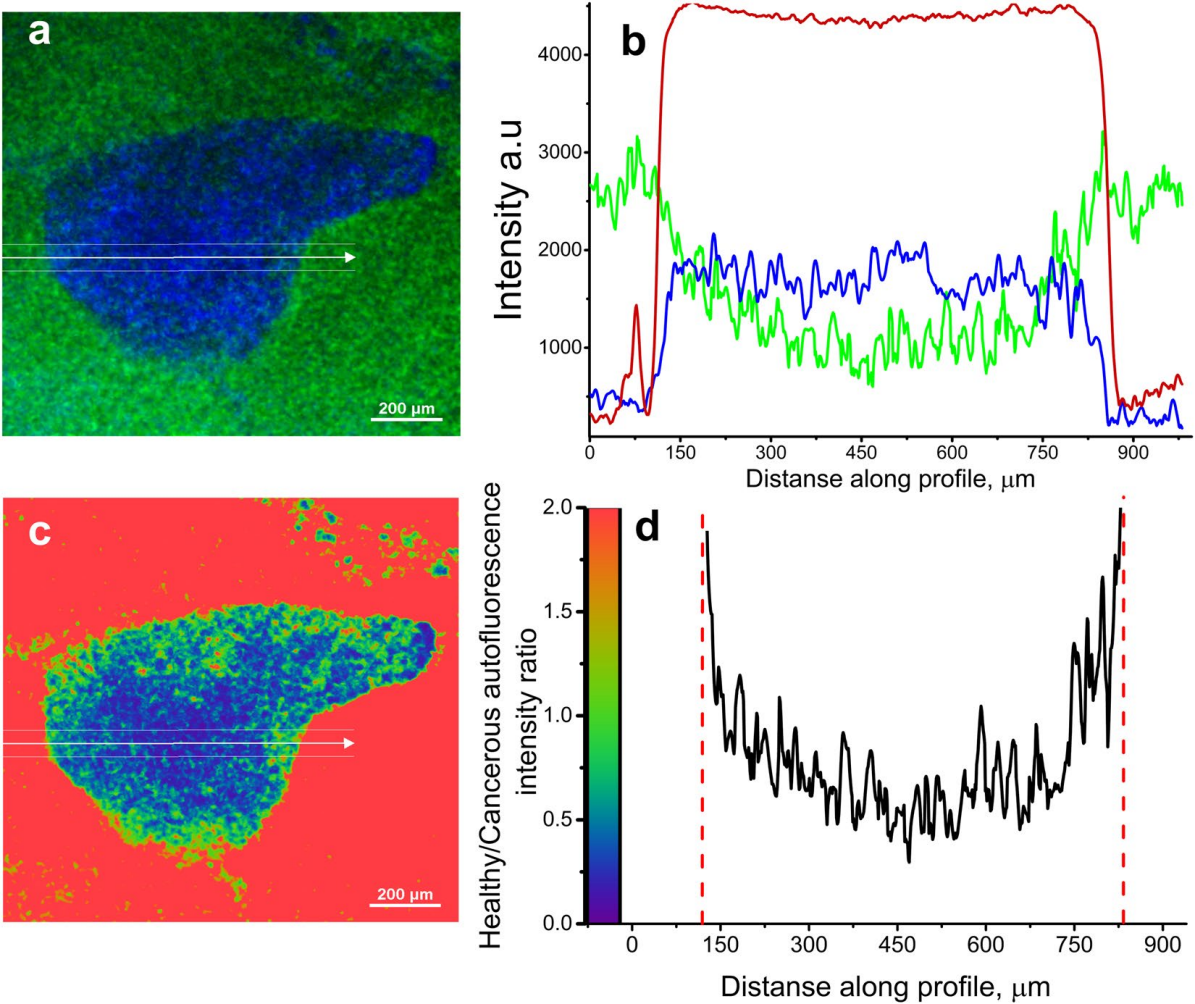
Spectrally unmixed images of lung cancer in human lung tissue modeled by OncoCilAir™. (**a**) tumor (blue) in healthy tissue (green), white arrow shows line along which intensity and ratiometric profiles were constructed. White lines indicate the segment margins; (**b**) intensity profile determined for mRFP component – red line; healthy tissue autofluorescence – green line; tumor – blue line. (**c**) ratiometric image with color code as shown in (**d**), (**d**) ratiometric profile (black line) along segment shown as white arrow in c). The intensity ratio obtained by dividing intensity of healthy tissue by cancerous tissue. In ratiometric profile red dashed lines is tumor edge determined from mRFP intensity profile.

The autofluorescence specificity for cancer cells were found to be in robust correlation with fluorescence from mRFP-expressing cells, further confirming health-dependent autofluorescence in this lung tissue model. Next, we built intensity profiles by plotting intensity values along a line segment in an image (Fig. 6a and b).

Intensity of cancerous tissue determined in the center of tumor body, on average, was twice as weak as autofluorescence from healthy tissue. Remarkably, autofluorescence signals inside tumors were not changing abruptly as observed for the mRFP profiles. The innate intensity profiles for autofluorescence corresponding to healthy tissue inside tumors were parabolic with a minimum in the tumor center while the autofluorescence intensity profiles of cancerous tissue inside tumors showed correlation with mRFP profiles. Next, we calculated the intensity ratio of fractions corresponding to healthy and cancerous tissue (Fig. 6c and d). Ratiometric images further demonstrated that, beginning from the tumor edge, autofluorescence decreases up to three-fold over 150 um. This suggests that metabolism of tissue inside cancer could be at an intermediate state that is in line with expected tumor influence on their microenvironment^71,72^. It should be noted that in the present study we did not observe autofluorescence modulation of fibroblast most likely because it has no direct contact with healthy tissue and tumor. While PET membrane is required to build the OncoCilAir™ model, it prevents possible direct tumor interaction with fibroblasts. In the near future, replacing the membrane with more permissive components, such as hydrogels or human based scaffolds, should eventually allow studying not only tumor-fibroblast interaction but possibly diffusion of cancer cells into the blood flow.

Ability to study metabolically related autofluorescence heterogeneity at an early stage of cancer development might help in the prevention of after surgery cancer remission because the interface between tumor and healthy tissue could be accessed accurately. For this purpose, the model could be further improved by incorporating tumor derived fragments directly into the airway epithelium to better represent the cell heterogeneity observed in patients’ tumour^73^. Moreover, autofluorescence could be monitored in preclinical development and validation of effective anticancer strategies on OncoCilAir™ to assess whether bronchoscopy is an efficient method to monitor anti-cancer strategies in patients.

## Conclusions

Here, we have demonstrated the unique ability of the OncoCilAir^TM^ lung cancer model to reproduce health tissue-dependent autofluorescence response typically observed *in vivo*. One of the most significant advantages of making use of this model is that all of its components are of human origin and specific to individual patients. The ability to develop novel autofluorescence-based tools in a personalized manner while also excluding laborious biopsy will undoubtedly boost autofluorescence bronchoscopy. Another advantage is the possibility of performing cancer development studies at the very earliest stages, usually inaccessible via conventional bronchoscopy. In addition, application of label-free autofluorescence methods for lung cancer with the OncoCilAir™ model conveniently allows monitoring under different conditions (e.g., drugs) without introducing fluorescent probes. On the contrary, fluorescent probes such as folate-fluorescein isothiocyanate^74^, indocyanine green^75^, $\gamma$-glutamyl hydroxymethyl rhodamine green^76^ which are designed for enhancing visualization of small tumors can be tested in close to actual conditions mimicked by OncoCilAir™.

Our findings provide evidence for the spectral response from healthy tissue that is in perfect agreement with the results previously reported from endoscopic autofluorescence bronchoscopy in patients. Moreover, autofluorescence is strongly tissue health-state dependent, and it exhibits spectrally different responses. Notably, in noncancerous tissue intensity is two-fold more intense than in cancerous. We showed that this contrast could be adequately understood by differences in amounts of endogenous fluorophores in tumors. Remarkably, we found that intrinsic autofluorescence of healthy tissue at the edges of tumors decays in the direction of the main body of tumors. Modulation of autofluorescence inside tumors indicates that our human lung tissue model, OncoCilAir™, replicates tumors’ ability to influence surrounding healthy tissues.
