# Author Correction: Health state dependent multiphoton induced autofluorescence in human 3D *in vitro* lung cancer model

**DOI:** 10.1038/s41598-018-24996-7

**Published:** 2018-04-24

**Authors:** Vasyl Kilin, Christophe Mas, Samuel Constant, Jean-Pierre Wolf, Luigi Bonacina

**Affiliations:** 1 GAP, University of Geneva, 22 chemin de Pinchat, CH-1211 Geneva 4, Switzerland; 2 OncoTheis Sàrl, 18 chemin des aulx, CH-1228, Plan-les-Ouates, Geneva, Switzerland; 3 Epithelix SAS, 219 Rue Laszlo Biro, 74160 Archamps, France

Correction to: *Scientific Reports* 10.1038/s41598-017-16628-3, published online 24 November 2017

The original version of this Article contained a typographical error in the spelling of the author Jean-Pierre Wolf, which was incorrectly given as Jean-Pier Wolf. This has now been corrected in the PDF and HTML versions of the Article.

